# Motivational Modulation of Self-Initiated and Externally Triggered Movement Speed Induced by Threat of Shock: Experimental Evidence for Paradoxical Kinesis in Parkinson’s Disease

**DOI:** 10.1371/journal.pone.0135149

**Published:** 2015-08-18

**Authors:** Louise M. McDonald, Harry J. Griffin, Aikaterini Angeli, Mariam Torkamani, Dejan Georgiev, Marjan Jahanshahi

**Affiliations:** Sobell Department of Motor Neuroscience and Movement Disorders, UCL Institute of Neurology, London, United Kingdom; Duke University, UNITED STATES

## Abstract

**Background:**

Paradoxical kinesis has been observed in bradykinetic people with Parkinson’s disease. Paradoxical kinesis occurs in situations where an individual is strongly motivated or influenced by relevant external cues. Our aim was to induce paradoxical kinesis in the laboratory. We tested whether the motivation of avoiding a mild electric shock was sufficient to induce paradoxical kinesis in externally-triggered and self-initiated conditions in people with Parkinson’s disease tested on medication and in age-matched controls.

**Methods:**

Participants completed a shock avoidance behavioural paradigm in which half of the trials could result in a mild electric shock if the participant did not move fast enough. Half of the trials of each type were self-initiated and half were externally-triggered. The criterion for avoiding shock was a maximum movement time, adjusted according to each participant’s performance on previous trials using a staircase tracking procedure.

**Results:**

On trials with threat of shock, both patients with Parkinson’s disease and controls had faster movement times compared to no potential shock trials, in both self-initiated and externally-triggered conditions. The magnitude of improvement of movement time from no potential shock to potential shock trials was positively correlated with anxiety ratings.

**Conclusions:**

When motivated to avoid mild electric shock, patients with Parkinson’s disease, similar to healthy controls, showed significant speeding of movement execution. This was observed in both self-initiated and externally-triggered versions of the task. Nevertheless, in the ET condition the improvement of reaction times induced by motivation to avoid shocks was greater for the PD patients than controls, highlighting the value of external cues for movement initiation in PD patients. The magnitude of improvement from the no potential shock to the potential shock trials was associated with the threat-induced anxiety. This demonstration of paradoxical kinesis in the laboratory under both self-initiated and externally-triggered conditions has implications for motivational and attentional enhancement of movement speed in Parkinson’s disease.

## Introduction

Bradykinesia, or slowness of movement, is one of the main symptoms of Parkinson’s disease (PD) and has a negative impact on the quality of life of patients [1]. People with PD can sometimes overcome their bradykinesia and move normally for a short time. This is known as paradoxical kinesis (PK). Some instances of PK are dramatic, such as fleeing from a fire [2], while others are more mundane, such as catching a ball [3]. A better understanding of PK could help pinpoint the nature of movement deficits in PD and contribute to better therapeutic strategies.

PD patients who experience bradykinesia have problems with planning, initiating and executing movements [4]. The most obvious manifestation of bradykinesia is a slow, shuffling gait with reduced stride length, but all movements, including arm movements and speech, are affected. Freezing, a temporary blocking of movement despite the intention to move, can also occur and can affect gait, arm movements and speech. Dopamine replacement therapy can reduce bradykinesia, but often does not alleviate it completely, especially as PD progresses.

Paradoxical kinesis was first described by Souques [5] who reported on severely bradykinetic patients who could sometimes move normally. One patient could climb stairs but struggled to walk in other circumstances. Luria [6] found that his PD patients could only walk across the floor when pieces of paper were placed on it ‘to stimulate each step’, but could climb ladders. Purdon-Martin [7] showed that bradykinetic parkinsonian patients could walk at normal speed over transverse lines, placed step-length apart. PK in emergency situations has also been reported, most notably during the L’Aquila earthquake in 2009. All 14 parkinsonian patients interviewed after the earthquake had been able to get up during the night and flee their homes and many helped others to escape [8].

Experimental studies on PK suggest that motivation may be an important factor. Three out of four parkinsonian patients were able to improve on their best arm movement speed when offered sixpence or a packet of cigarettes [9]. PD patients trained on an arm movement task with smiley and sad faces as feedback improved their movement speed to reach the same range as controls [10]. People with PD and healthy controls moved 7% faster in an ‘urgent’ condition compared to an externally cued condition when asked to press a button to stop a ball rolling off a slope [11]. In this study task difficulty was adjusted for individual participants, allowing a PK-like increase in movement speed to be demonstrated in healthy controls. In another study, speech duration increased by 49% in PD patients who talked about experiences of anger or fear, which are associated with high physiological arousal [12].

External stimuli can also increase movement speed in PD patients, particularly compared to self-initiated movements. PD patients were 54% slower than controls on a self-initiated (SI) button press, but both groups improved by 16% when the movement was triggered by an external cue (ET) [11]. When asked to press a button to stop a cartoon character on a screen being run over by a car, PD patients were 50% slower than controls in a SI condition and improved by 4–8% in an ET condition [13]. In a task that involved reaching for a stationary ball (SI) and a ball rolling down a ramp (ET), PD patients were 59% slower than controls in the SI condition but matched the controls’ performance in the ET condition [14].

It should also be noted that motivational enhancements of movement speed have been described in healthy individuals. Ballanger et al. [11] found similar enhancements of movement speed in PD patients and healthy controls in a ball-catching task, where both groups were able to move faster than their previous maximum speed in an ‘urgent’ condition. Ballanger et al. [11] suggested that reports of motivational increases in movement speed in healthy individuals in PK experiments are rare because most PK studies tend to have an upper limit on movement speed in their tasks, which affects healthy controls, but not PD patients. Schmidt et al. [15] found greater grip force production in healthy participants when higher monetary incentives were offered. There have also been a number of anecdotal reports of people showing extraordinary strength (e.g. lifting a car to rescue someone), which may be examples of motivational enhancement of movement in healthy individuals [16]. We would therefore suggest that it is reasonable to expect an increase in movement speed in healthy controls as well as PD patients in the current study.

In the present study we were interested in investigating the effects of motivation as well as external cuing on movement speed in PD and healthy controls. Therefore, we developed a task that incorporated both motivational and external cueing components. The task required participants to react to stimuli presented on the screen by releasing a ‘home’ key and moving to press another ‘response’ key as fast as they could. At the start of each trial there was a warning signal indicating whether or not they could receive a mild electric shock if they did not move fast enough. They performed two types of movements—self-initiated (SI) and externally-triggered (ET) and received feedback at the end of each trial as to whether they had moved fast enough. The criterion for receiving a shock was the threshold movement time that was set for each individual on each trial using a staircase tracking procedure, based on each participant’s performance on previous trials. Our hypotheses were first, that both people with PD and healthy controls would move faster under the threat of a mild electric shock. Second, we expected people with PD to perform at similar speeds to healthy controls in the externally-triggered condition, but that controls would move faster than PD patients in the self-initiated condition.

## Methods

### Participants

The study was approved by the Joint Ethics Committee of the Institute of Neurology and the National Hospital for Neurology and Neurosurgery. All participants were screened for dementia (see below) and none were demented and all had capacity to consent. Informed consent was obtained from all participants in writing before starting the study. 16 PD patients and 17 controls were recruited. One PD patient did not perform the task correctly and another scored less than 25 on the MMSE. They were therefore excluded. One control did not perform the task correctly and two others had missing data on the main task due to technical problems. Data from 14 PD patients (10 male, 12 right-handed) and 14 (10 male, 13 right handed) healthy age-matched controls are reported. The demographic and clinical details of the samples are presented in Table 1. All patients had a clinical diagnosis of PD according to the UK Brain Bank criteria [17]. The patients had mild to moderate PD according to Hoehn & Yahr rating scale [18] (Table 1). The Mini Mental State Examination (MMSE [19]; the Beck Depression Inventory (BDI [20]; were respectively used to screen for dementia and depression. None of the patients were cognitively impaired (MMSE score <26) or clinically depressed (BDI score > 18). The National Adult Reading Test (NART) was used to obtain an estimate of pre-morbid IQ and to match the two groups.

**Table 1:** Demographic data and scores on tests of cognition, executive function and mood scales for controls and patients with Parkinson’s disease (PD). The data are means (±SEM).

	Control	PD	*p*
N	14	14	-
Gender	10 male	10 male	1.00
Age	68 (1.69)	65.00 (1.95)	.26
MDS-UPDRS (Motor part)	-	35.31 (3.37)	-
Medication (L-DOPA equivalent)	-	761.94(150.27)	-
Hoehn and Yahr stage	-	2.06(0.07)	-
Education (years)	15.64 (0.88)	14.86 (0.60)	.47
Handedness (EHI)	72.29 (10.54)	58.93 (14.26)	.46
BDI (total)	5.04 (1.16)	11.82 (1.96)	.006
Non-somatic (questions 1–14)	3.11 (1.09)	6.29 (1.46)	.09
Somatic (questions 15–21)	1.93 (1.07)	5.50 (0.60)	< .001
MMSE	29.29 (0.27)	29.43 (0.23)	.67
NART IQ	122.29 (1.64)	121.29 (1.33)	.64

MDS UPDRS = Movement Disorder’s Society Unified Parkinson’s Disease rating Scale; EHI = Edinburgh Handedness Inventory; BDI = Beck Depression Inventory; MMSE = Mini Mental State Examination; NART IQ = National Adult Reading Test Intelligence Quotient.

None of the control participants had any neurological or psychiatric illnesses, head injury, and alcohol or drug abuse. The control and PD groups were matched in terms of age, estimates of premorbid IQ (NART), global cognitive ability (MMSE), years of education, and degree of right-handedness. While as a group the patients scored significantly higher on the BDI measure of self-reported depression than the controls, none scored in the severely depressed range. Furthermore, more detailed analysis showed that relative to the controls, the PD patients had higher scores on the somatic items of the BDI which somewhat overlap with PD symptoms but not the cognitive/affective component of depression (Table 1).

### Equipment

The program was run on a Dell Optiplex GX520 (3.0 GHz) computer running MATLAB R2010a. A custom-built button box with 2 buttons was used to record responses. The buttons were 2.5cm in diameter and 15cm apart (measured from the centre of each button). Both buttons were directly in front of the participant, with the ‘home’ key being nearer and the ‘response’ key further away. Shock was delivered using a Digitimer Constant Current Stimulator Model D57A set to a pulse width of 2000μs and 400V. Shock ranged from 0.3 to 3.8mA according to each participant’s threshold and was delivered in a 1s train of 50ms pulses via an electrode with metal contacts attached to the forearm just above the wrist with a Velcro strap.

### Shock Avoidance Behavioural Paradigm

**Fig 1:**
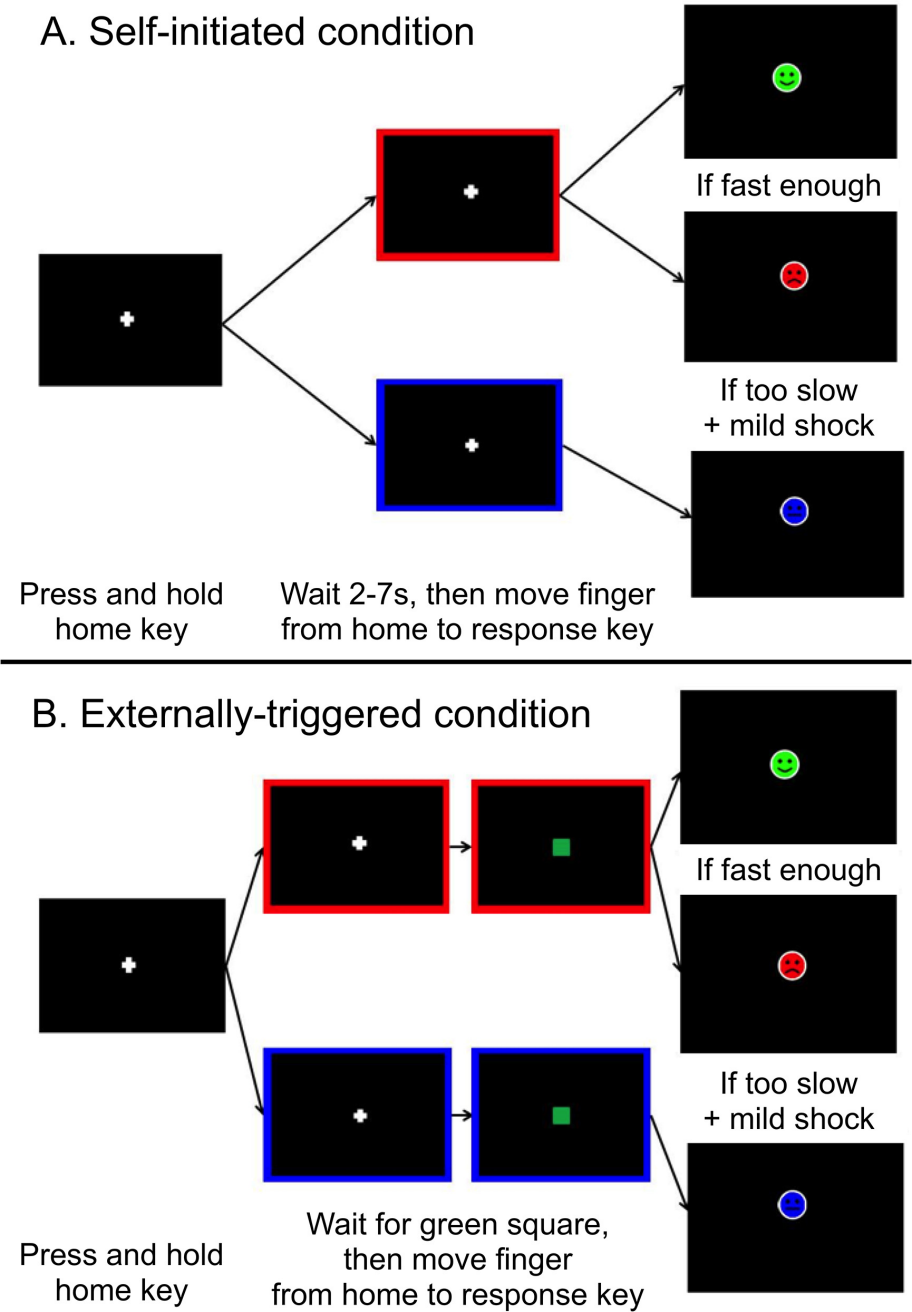
The Shock Avoidance Behavioural paradigm with self-initiated (A) and externally-triggered (B) conditions.

The shock level was calibrated for each individual, starting at 0 and moving up in steps of 0.2mA until the participant reported the shock as being unpleasant but not painful. After this shock calibration procedure, participants were provided with verbal and on-screen instructions about the shock avoidance behavioural paradigm. Fig 1 shows the sequence of events on the screen during the different types of trials of the task. When a white fixation cross appeared in the middle of the black screen, participants were required to fixate on the white cross and press down and hold the near ‘home’ key on the button box. After a variable delay of 2–6s, a red or blue border appeared around the edge of the screen. A red border indicated a potential shock (PS) trial and warned the participant that there could be a shock delivered on that trial if they did not move fast enough (at individually set criterion for movement speed, defined below) from the near to the far button. A blue border signalled a no potential shock (NPS) trial and that there would be no shock on that trial. On self-initiated (SI) trials (Fig 1A), participants were then asked to wait for a variable time of 2–7s before moving as fast as they could from the ‘home’ to the ‘response’ button. In externally-triggered (ET) trials (Fig 1B), participants were instructed to release the ‘home’ key and press the response button as fast as possible following the presentation of the green square (external signal) on the screen. After each trial, the participant received feedback, lasting for 600 ms, via a cartoon face—a smiley or a sad face on PS trials or a neutral face on NPS trials—as to whether they had moved fast enough. A shock was delivered immediately after the response button press simultaneously with the ‘sad’ face if participants failed to move fast enough on a PS trial.

There were eight blocks of 40 trials each. Half of the blocks were SI and half were ET. In each block, all trials were either SI or ET. The first block was SI and the block types then alternated, with the delay between the border change and appearance of the green square to trigger movement in each ET trial being yoked to the delay between the border change and start of the participant’s movement in the previous SI block. Within each block, 20 PS and 20 NPS trials were delivered in a pseudo-random order, so that there could be no more than 3 consecutive trials of one type. The criterion for shock delivery was a threshold movement time, which was set for each trial for each participant using a staircase tracking procedure, with an initial value of 300ms, which reduced by 20ms every time shock was successfully avoided and increased by 50ms every time a participant received a shock.

Movement times (MTs) were measured in both the SI and ET conditions and reaction times (RTs) were measured in the ET condition. MTs were measured as the time between the participant releasing the home button and pressing the response button. In the ET condition, RTs were measured as the time between presentation of the green square ‘go’ stimulus and release of the home button by the participant. In the SI blocks, the waiting time (WT) was calculated as the difference between the time of presentation of the warning stimulus and the time of release of the home key by the participant. The difference in MT between NPS and PS trials (MT improvement) was used for correlational analysis.

### Procedure

All PD patients were tested ON medication. The Movement Disorders Society-Unified Parkinson’s Disease Rating Scale (MDS-UPDRS) rating scale [21] was used to assess the severity of the motor symptoms at the time of the experiment by an experienced neurologist (AA). All participants rated their anxiety on a 0 (not at all anxious)-10 (very anxious) scale before starting the shock avoidance paradigm and immediately after it ended. The Edinburgh Handedness Inventory [22] was used to quantify the degree of right-handedness. The screening measures (MMSE, BDI, NART) were completed after the shock avoidance paradigm.

### Analysis

All statistical analyses were completed with SPSS(v19). Demographic data were compared between the control and PD groups using independent groups’ t-tests for interval data and χ^2^ for categorical data. Anxiety before and after the experiment was analysed using a repeated measures ANOVA with Time of Assessment (Before vs. After) as within-subject factor and Group (PD vs. controls) as between-subject factor.

Parametric tests were used because the raw MT and RT data were normally distributed and had homogeneous variances. Data, which represented counts (i.e. number of shocks received) were analysed using the χ^2^ test.

MTs and RTs were summarised into mean values for each individual for each combination of Condition (Self-initiated (SI) versus Externally-triggered (ET)) and Trial Type (Potential Shock (PS) versus No Potential Shock (NPS)). MT was the main outcome measure and was analysed using repeated measures ANOVA with Condition (SI or ET) and Trial Type (PS or NPS) as the within-subjects factors and Group (control or PD) as the between-groups factor. RT for ET blocks was analysed using a repeated measures ANOVA with Trial Type (PS or NPS) as the within-subject factor and Group (control or PD) as the between-groups factor.

Correlations between each participant’s average MT improvement between NPS and PS trials and their anxiety ratings were calculated using Pearson’s correlation coefficient.

## Results

The control and PD groups were well matched in terms of demographic variables, and estimates of premorbid IQ. There was no significant differences between the groups on any measure except for BDI, where the PD group scored higher (t(26) = -2.99, p = .006, r = .491). However, when BDI scores were divided into non-somatic (questions 1–14) and somatic (questions 15–21) items [23], there was no difference between the groups on non-somatic items (t(26) = -1.75, p = .09, r = .313), and the PD group scored significantly higher on somatic items (t(18.7) = -5.39, p < .001, r = .715). See Table 1 for details.

Anxiety was rated by the participants on a scale of 0 (low) to 10 (high) before and after the shock avoidance paradigm. Before the paradigm, controls reported a mean anxiety of 1.43 (SD = 1.83) and PD patients reported a mean anxiety of 2.07 (SD = 2.24). After the shock avoidance paradigm, controls reported a mean anxiety of 1.07 (SD = 1.87) and PD patients reported a mean anxiety of 1.71 (SD = 1.83). An ANOVA on anxiety ratings before and after the shock avoidance paradigm for controls and PD patients showed no effect of Group (F(1,26) = 1.09, p = .304, *η* ^*2*^ = .304) or time of assessment (before vs. after shock avoidance) (F(1,26) = 0.78, p = .384, *η* ^*2*^ = .029) and no significant interaction (F(1,26) <1, p = 1.00, *η* ^*2*^ = .000).

### Impact of threat of shock on speed of movement initiation and execution

**Fig 2:**
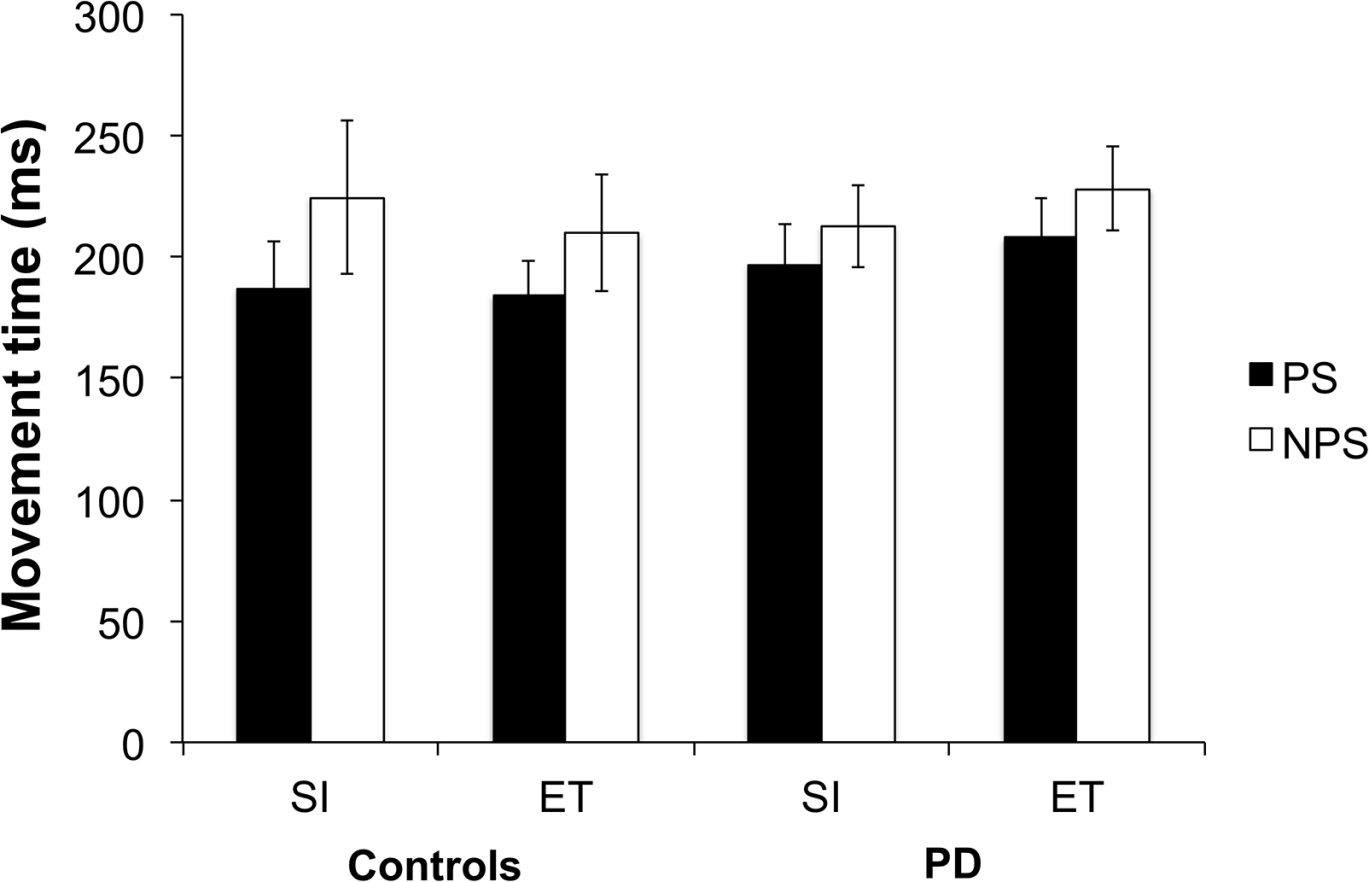
Movement time (ms) for controls and patients with Parkinson’s disease (PD) in self-initiated (SI) and externally-triggered (ET) conditions for the potential shock (PS) and no potential shock (NPS) trials.

Both healthy controls and PD patients demonstrated increased movement speed, moving faster when threatened with shock in both the SI and ET conditions (see Fig 2). The Group × Condition × Trial Type ANOVA on MT produced a significant main effect of Trial Type (F(1, 26) = 12.11, p = .002, *η* ^*2*^ = .331), with MTs on PS trials being significantly faster than on NPS trials. This improvement of MTs with threat of shock was observed across both groups and movement speed increased by 8–9% for PD patients and 13–15% for the controls. There were no other significant main or interaction effects.

**Fig 3:**
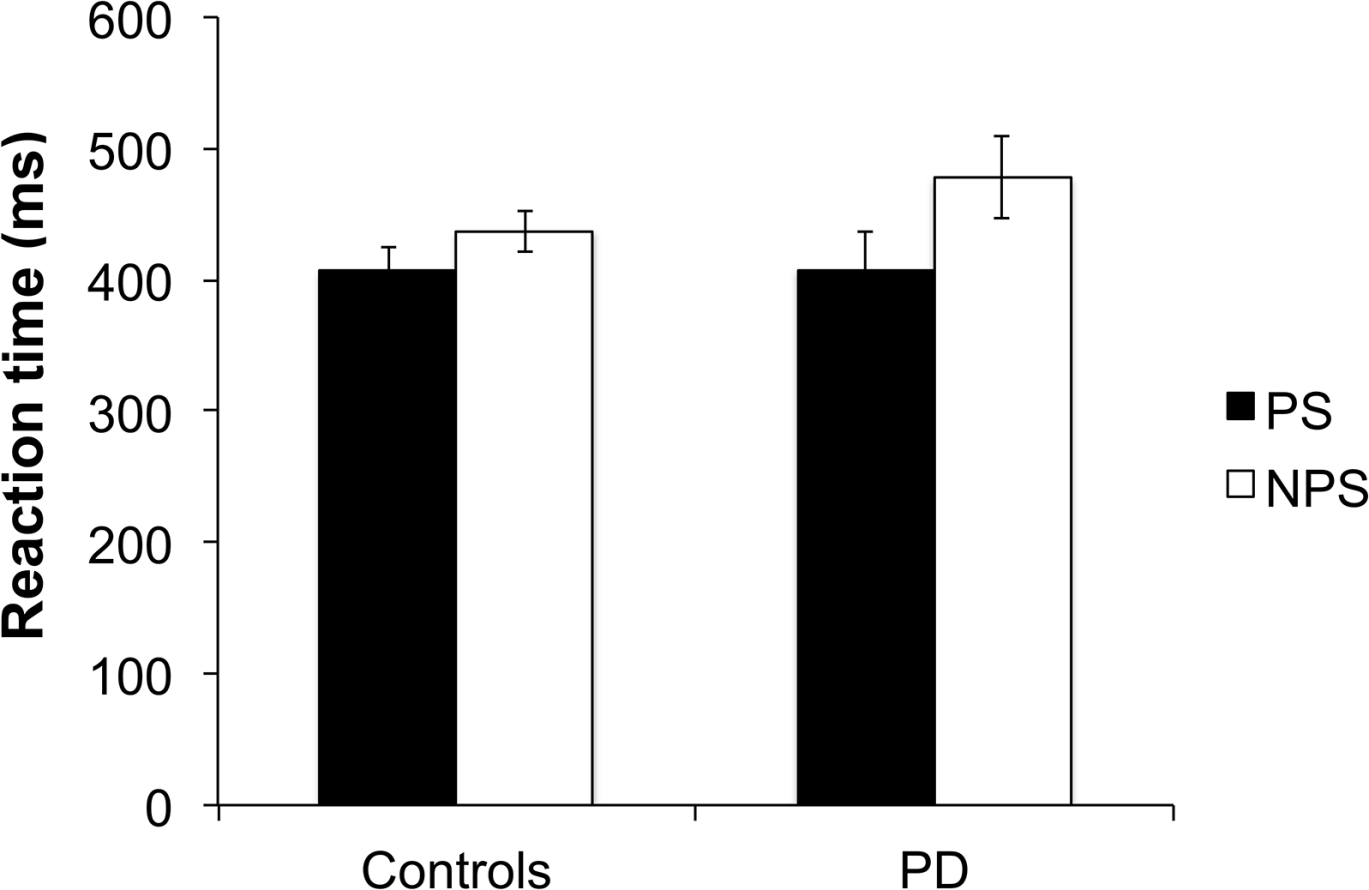
Reaction time (ms) for controls and patients with Parkinson’s disease (PD) in the externally-triggered condition in potential shock (PS) and no potential shock (NPS) trials.

The analysis of RTs was done for the ET trials, as there is no true RT measure in SI trials. The mean RTs for the PS and NPS trials of the ET condition for the controls and PD patients are shown in Fig 3. A two-way repeated measures ANOVA on Trial Type (Shock versus No Shock) and Group (Control versus PD) showed a significant effect of Trial Type (F(1,26) = 40.49, p < .002, *η* ^*2*^ = .609), no significant effect of Group (F(1,26) = .39, p = .540, *η* ^*2*^ = .015), but a significant Group × Trial Type interaction (F(1,26) = 6.42, p = .018, *η* ^*2*^ = .198). Simple main effects tests on the effect of Trial Type for each Group showed that both controls (F(1,13) = 10.01, p = .007, *η* ^*2*^ = .435) and PD patients (F(1,13) = 31.23, p < .0001, *η* ^*2*^ = .706) had significantly faster RTs in the ET condition on PS trials compared to NPS trials. Thus the threat of shock significantly reduced RT for PD patients and controls. However, this improvement in RTs with threat of shock was significantly greater in PD patients (mean = 70.31, SD = 47.08) than in healthy participants (mean = 30.27, SD = 35.78) (t(26) = 2.53, p = .018, r = .403) (Fig 3).

There was no significant difference in the WT for the SI blocks between PD (mean WT = 1907.93, SD = 1353.23) and healthy controls (mean WT = 1933.14, SD = 1176.81) (p = .946). As expected, across both groups, the waiting time was shorter in the PS (mean WT = 1851.74, SD = 1116.04) compared to NPS trials (mean WT = 1989.14, SD = 1204.81) (F(1,13) = 18.65, p < .0001, *η* ^*2*^ = .400). The Group × Trial Type interaction for WT was not significant F(1,26) = 0.73, p = 401, *η* ^*2*^ = .025).

Although the PD patients chose a slightly higher average shock level (mean = 1.59mA, SD = 0.52) than the control participants (mean = 1.47mA, SD = 1.18), there was no significant difference between the groups (t(17.86) = -0.343, p = .735, r = 0.065). Application of the staircase tracking procedure adjusted the MT shock criterion individually for each participant in the course of the experiment, to ensure that all participants received approximately the same number of shocks. The mean MT criterion for delivery of shock was 175.32ms (SD = 74.11) for control participants and 198.27ms (SD = 67.11) for PD patients. These were not significantly different (t(26) = -0.859, p = .398, r = .160). Controls and PD patients received similar numbers of shocks during the experiment (χ^2^ = 3.56, p = .47, r = .356), with both groups receiving shocks on 20% of PS trials.

The magnitude of improvement of MTs from NPS to PS trials positively correlated with anxiety ratings after the shock avoidance paradigm (r(28) = .47, p = .01) as well as with the shock criterion (r(28) = .487, p = .009). The raw data of the experiment can be found in the Supporting Information (S1 File).

## Discussion

PD patients had a faster MT in the potential shock (PS) than the no potential shock (NPS) condition, demonstrating paradoxical kinesis (PK) in an experimental setting. Healthy controls also showed an increase in movement speed from the NPS to PS condition. There was no difference in the increase in movement speed between SI and ET conditions. In addition, both PD patients and healthy controls had faster RTs in the ET condition when threatened with mild electric shock; this improvement of RTs was greater for PD patients than controls.

Bradykinesia in PD has been described as “insufficient motor energy” and it has been demonstrated that PD patients could move faster but ordinarily chose not to do so, because of the energetic cost entailed by moving at normal speed [10]. Our results confirm that when provided with appropriate motivational contexts, in the form of shock avoidance, patients with PD can speed up their movements.

In the current study, both PD patients and controls improved their MT when faced with the threat of mild electric shock. Across both the SI and ET conditions, the shock-induced MT improvement for the controls (13–15%) was slightly greater than the improvement in PD patients (8–9%), although this difference was not significant. A number of other studies have addressed the effects of motivation on movement. Two general forms of motivation have been studied: the motivation to earn a reward and the motivation to avoid a punishment (as in our study). The majority of previous studies have examined the motivation to earn a reward. Hall [9] reported increased movement speed in 3 out of 4 parkinsonian patients by offering sixpence or a packet of cigarettes and Mazzoni et al. [10] trained PD patients to perform an arm movement as fast as controls by reinforcing fast or slow movements appropriately with smiley or sad cartoon faces. Kojovic et al. [24] found faster movement initiation in medicated and unmedicated PD patients in a simple reaction time task in anticipation of monetary reward for faster performance. Moustafa et al. [25] found that medicated PD patients were better at speeding up response times to earn a reward, while unmedicated PD patients were better at slowing their responses to earn a reward in a task where participants had to choose between immediate or delayed responses to maximise rewards. Only Shiner et al. [26] have studied the motivation to earn a reward and the motivation to avoid a punishment in the same paradigm in PD patients tested off medication. Participants were asked to move as fast as possible to earn money or avoid a shock in a self-initiated task. They found that PD patients improved more on shock trials than on monetary incentive trials compared to controls. This suggests that the impact of the two forms of motivation is not behaviourally identical, although there are common features. Our findings that PD patients (and controls) could speed up their movements further to avoid punishment, despite already having been asked ‘to move as fast as they can’, are consistent with previous studies. However, the results of Shiner et al. [26] suggest a difference between these two kinds of motivational influence, monetary incentive versus shock avoidance, on movement speed, which requires further examination in future studies.

The finding of improved movement speed in healthy controls as well as PD patients is consistent with Ballanger et al. [11], who asked participants to press a button to stop a ball rolling off a slope. Mir et al, [27], Kojovic et al. [24] and Shiner et al. [26] also found that controls could improve on their maximum movement speeds when motivated by earning a reward or avoiding punishment. In a grip force production task, healthy controls were able to increase their grip force with the prospect of higher monetary rewards [15]. This suggests that motivational modulation of movement speed and force is a feature of normal motor function and present in healthy controls as well as preserved to an extent in PD patients.

There was also a reduction of RTs in the PS condition of the ET blocks for both PD patients and healthy controls. This speeding up of RTs was however greater in magnitude for PD patients than the healthy controls, possibly due to a ‘floor effect’ for the controls It is possible that altered time estimation contributes to an extent to the observed improvement of RTs with threat of shock. However, this proposal is not consistent with previous studies on time estimation under stress, which found that stress or anxiety lengthen time estimation [28, 29]. Nevertheless, threatening events are considered to speed up the rate of an “internal clock”, such that when prompted to produce a response under threatening circumstances individuals make responses faster than they might otherwise and perceive more time as having elapsed than actually has [30]. Enhanced attention is another potential mechanism for the observed speeding of RTs with threat of shock. Paying attention to making larger movements has been shown to improve movement speed in PD [31], so it is possible that participants focusing their attention on producing faster MTs in the PS condition in the current study also improved RTs. The greater magnitude of improvement of RT with motivation to avoid shock in the ET condition for the PD patients, but the larger improvement of MTs across SI and ET conditions for the healthy controls, is consistent with previous findings that relative to controls, PD patients are more impaired on SI than ET movements [32].

Operant conditioning does not explain the faster MTs and RTs in the PS condition, as the MT criterion for shock delivery was altered in every trial using a staircase tracking procedure, so conditioning to a consistent criterion would not have taken place. Even in a PS trial, there was only a 20% chance of receiving a shock, suggesting that any conditioning that might have taken place would have been more likely to be in the direction of non-occurrence of shock and cannot provide an alternative explanation for faster MTs in this condition. The 80% likelihood of non-occurrence of shock is the same probability that is employed for conditioning in probabilistic learning experiments [33], so was the more likely contingency to be learned here.

The magnitude of MT improvement from the no potential shock to the potential shock trials was positively correlated with anxiety scores after the shock avoidance paradigm. It is possible to maintain and even improve task performance when anxiety is increased by the threat of shock, as shown by Robinson et al. [34], who found sustained performance and enhanced negative bias when participants completing an emotional Stroop task were threatened with shock.

Motivational modulation of movement speed, as in the current study, suggests that the limbic system can influence the motor system. Such motivational modulation of movement could be mediated via the ventral tegmental area (VTA) and the nucleus accumbens (NA) which form a limbic-motor interface with the vental pallidum (VP), as the medial sections of these structures (NAshell, VPm, VTA) receive limbic input; whereas the lateral sections (NAcore, VPl, substantia nigra) are connected to the motor system. There are also afferents from the VTA to the limbic system and efferents from the limbic system to the cingulate cortex, via the thalamus [35]. The VTA together with the substantia nigra, is involved in the relationship between stimuli and movement [36] and the cingulate cortex has been activated in studies demonstrating PK in PD patients and similar motivational enhancement of movement in controls [37–39]. In fMRI studies of the effects of monetary incentive on grip force production [15, 40], activation in the VP and ventral striatum was correlated with the behavioural effects of incentives on grip force.

### Limitations and future directions

We expected to find faster MTs in the ET version of the task, compared to the SI version, particularly for the PD patients, but the MTs were similar in both conditions. This may be a genuine finding, but it may also be a consequence of the warning signal indicating the start of both the SI and ET trials effectively reducing the extent of self-initiation in the SI trials. This possibility needs to be checked in future studies with an SI task without a warning signal before each trial, although there are considerable practical difficulties in designing such a task.

There was no difference in MT between controls and PD patients. This may have been due to the PD patients being tested while on dopaminergic medication which effectively controlled their motor symptoms. It has been proposed that tonic levels of dopamine have an energizing effect and control response vigor [41]. If response vigor is modulated by striatal dopamine, then it would be of interest to determine whether the speeding of MT with threat of shock is different when patients are tested off medication. Future investigation of motivational modulation of movement speed in subgroups of PD patients with motivational deficits due to apathy or depression would also be of interest.

A novel feature of our paradigm was testing for motivational modulation of movement speed in both ET and SI conditions. There is substantial evidence that bradykinetic PD patients have fewer problems when movements are ET than SI and move faster in ET tasks (e.g. [13, 32]. Observations of PK in PD often include situations with external stimuli such as a fire or transverse lines [7] or catching a ball [3]. However, we found no difference in the threat of shock-induced improvement of MT, between ET and SI conditions for either PD patients or controls, suggesting that motivational modulation of movement speed occurs for both types of movement. It may be necessary for a motivational element to be present, such as the wish of avoiding shock, for motivational increases in movement speed to be present under SI conditions. Another possible explanation for the presence of motivational increases in movement speed in our SI task might be that the warning signal (the screen border change that signalled whether a trial was PS or NPS). A similar signal was present in the Shiner et al. [26] study—participants were instructed to wait for the disappearance of a cue—and is difficult to avoid, as participants need to be given a starting signal of some kind even in SI conditions. A warning signal significantly speeded up RT and MT in a button press task when given at intervals from 200–3200ms before the task start signal [42], so could have reduced the difference between SI and ET tasks in this paradigm. The potential effect of the warning signal should be tested in a version of this task without a warning signal preceding each trial.

### Conclusions

This study demonstrated PK in PD patients tested on medication and a similar motivational enhancement of movement speed in age-matched controls in a task where faster movements were motivated by the threat of mild electric shock. PK was demonstrated in both the ET and SI versions of the task and this may be the first experimental demonstration of PK in PD in a SI task. Such motivational modulation of movement speed suggests the possibility of limbic system influencing and interacting with the motor system. Further work is needed to extend the demonstration of PK in an SI task to an SI task without a warning signal and to test whether PK can be elicited in this task in PD patients off medication. Future investigation of the neural substrates of the observed motivational modulation of movement speed in this shock avoidance paradigm in healthy controls and patients with PD will clarify if the threat of shock induced improvement of MT is similarly mediated in the two groups. Such motivational modulation of movement speed in PD may have therapeutic applications to teach patients to overcome bradykinesia.

## Supporting Information

S1 File This file contains the raw data for every participant separately. RT = Reaction Time, MT = Movement Time, Exact time of: trial start, home key press and release, warning signal appearance, target key press, feedback and Inter Trial Interval (ITI).
(XLSX)
